# IMPLEMENTING AND TESTING THE EFFICACY OF A GERIATRIC GOALS OF CARE TOOL IN AN ACUTE HOSPITAL SETTING

**DOI:** 10.1093/geroni/igad104.2932

**Published:** 2023-12-21

**Authors:** Pallavi Samudrala, Brynn Sheehan, Erin Smith, Marissa Galicia-Castillo

**Affiliations:** Eastern Virginia Medical School, Norfolk, Virginia, United States; Eastern Virginia Medical School, Norfolk, Virginia, United States; Sentara Leigh Hospital, Norfolk, Virginia, United States; Eastern Virginia Medical School, Norfolk, Virginia, United States

## Abstract

Goals of care (GOC) discussions, which focus on patient values and preferences, are associated with improved outcomes for older adults, including lower hospital readmission rates, increased adherence to advanced care plans, and improved patient satisfaction. GOC discussions do not occur consistently and a reliable method of documentation has yet to be established. The Geriatrics Goals of Care (GGOC) tool was developed to easily review and document GOC discussions for geriatric patients (65 years and older) in the electronic medical record, focusing on pre-hospital function and hospitalization goals. The study objective was to evaluate the GGOC tool’s impact on 30-day hospital readmission. Using an experimental design, the GGOC tool was implemented in one hospital unit and a second comparative unit served as a control. The GGOC tool was administered to a total of 82 patients and 162 control unit patients received standard care (N= 244). A logistic regression of patient-level data revealed that the odds of having a hospital readmission within 30 days were approximately 65% lower for patients who received the GGOC tool compared to those in the control unit (OR = .344, SE = .40, p = .008, 95% CI [.156, .759]). The readmission rate for the unit utilizing the GGOC tool yielded a cost savings of $185,019.12. Applying the same readmission rate to the control unit, the expected cost savings was $365,625 for the control group. Results suggest that the GGOC tool may be effective for documenting GOC discussions, reducing the burden of readmissions with their associated costs.

